# P54 Evaluating capacity issues and safety of uveitis adolescent services at Moorfields Eye Hospital (MEH)

**DOI:** 10.1093/rap/rkac067.054

**Published:** 2022-09-28

**Authors:** Marcela Bohn, Harry Petrushkin

**Affiliations:** Moorfields Eye Hospital NHS Foundation Trust, London, United Kingdom; University College London Hospitals NHS Foundation Trust, London, United Kingdom; West Hertfordshire Teaching Hospitals NHS Trust, Watford, United Kingdom; Moorfields Eye Hospital NHS Foundation Trust, London, United Kingdom; Great Ormond Street Hospital, London, United Kingdom; University College London Hospitals NHS Foundation Trust, London, United Kingdom

## Abstract

**Introduction/Background:**

The uveitis Young Adults Clinic (YAC) was implemented at MEH in 2019, with the aim of facilitating the transition from children's to adults' services. It receives new patients mainly from paediatric uveitis clinics at MEH and Great Ormond Street Hospital (GOSH), both reference centres, but also from multiple other paediatric services across London and the UK. The patients are seen from the age of 16 to 21 (or more in special circumstances). As this clinic runs only for one session weekly, it has been facing capacity issues with overbooking and backlog of patients waiting to be booked.

**Description/Method:**

A service evaluation audit was performed with the objectives of quantifying the Uveitis YAC capacity issues and evaluating its safety during the period from December 2020 to December 2021.

1) Clinical capacity issues were quantified by checking clinic numbers per staff (new and follow-up patients) and comparing them to other uveitis clinics and the service’s template of 6 patients/doctor.

2) Clinic safety was analysed by:

- Checking the percentage of YAC patients that receive their hospital appointments within 25% of their intended follow-up period (25% follow-up delay target), following the Royal College of Ophthalmology key outcome measure (minimum standard of 85%).

- Quantifying the backlog of new patients waiting to be booked into clinic for more than 18 weeks (RTT referral to treatment time 18 weeks - minimum standard of 85%).

A total of 614 patient visits were identified during that time (with 202 unique patient IDs). The data sources used to extract the data were electronic medical records (PAS, Openeyes) and doctors' rota. Patient data were extracted in an automated way from EMR by a senior information analyst from the Service Improvement department. Data processing and analysis were done by two members of the clinical team independently with Excel and R.

**Discussion/Results:**

A total of 421/614 visits had available intended follow-up times at PAS. Of these, 246 patients (58%) were seen within 25% of their requested follow-up.

A total of 303 new patient appointment requests were retrieved from the YAC waiting list during that period (screenshot of first day/month). Of these, 94 were unique patients (patients stayed in the waiting list for more than one month). We excluded the patients added in the last month of December 2021 (total 18), as there was no way of checking when they left the waiting list. Out of the remaining 76 patients, 56.6% were not seen within 18 weeks of referral (43.4% were seen).

There were 56 clinic dates during the period December 2020 to December 2021. From the first clinic in December 2020 to the last in June 2021, 191 patients were seen in YAC (178 follow-ups and 13 new). The mean number of patients per doctor was 6.8. From July to December 2021, 213 patients were seen (198 F/U and 15 news). The mean number of patients per doctor was 8.5. When comparing to the adult uveitis clinic which happens at the same dates and times, in the first 6 months, the number of patients per doctor was 4.3, compared to 5.8 in the second semester.

The results show that the follow-up times are not reaching safety standards following RCO recommendations. New patient referrals are not being seen according to RTT standards. The number of patients per doctor exceeds service guidelines of 6 patients/doctor for complex uveitis patients.

**Key learning points/Conclusion:**

The YAC is a unique service that receives patients from major London uveitis services and works closely with UCLH Rheumatology Adolescent services to deal with complex and vulnerable cases. This audit shows that the clinic is working above this capacity and needs further support to be able to provide safe and high-quality care for adolescent patients. Possible improvements would involve increasing the clinical team, ideally with multidisciplinary members, and optimising scrutiny and booking of patients.

